# The Molecular Evolution and Functional Divergence of Lamprey Programmed Cell Death Genes

**DOI:** 10.3389/fimmu.2019.01382

**Published:** 2019-06-20

**Authors:** Xin Guan, Jiali Lu, Feng Sun, Qingwei Li, Yue Pang

**Affiliations:** Lamprey Research Center, Liaoning Normal University, Dalian, China

**Keywords:** PDCD family, evolution, apoptosis, lamprey, immune regulation

## Abstract

The programmed cell death (PDCD) family plays a significant role in the regulation of cell survival and apoptotic cell death. However, the evolution, distribution and role of the PDCD family in lampreys have not been revealed. Thus, we identified the PDCD gene family in the lamprey genome and classified the genes into five subfamilies based on orthologs of the genes, conserved synteny, functional domains, phylogenetic tree, and conserved motifs. The distribution of the lamprey PDCD family and the immune response of the PDCD family in lampreys stimulated by different pathogens were also demonstrated. In addition, we investigated the molecular function of lamprey PDCD2, PDCD5, and PDCD10. Our studies showed that the recombinant lamprey PDCD5 protein and transfection of the L-PDCD5 gene induced cell apoptosis, upregulated the expression of the associated X protein (BAX) and TP53 and downregulated the expression of B cell lymphoma 2 (BCL-2) independent of Caspase 3. In contrast, lamprey PDCD10 suppressed apoptosis in response to cis-diaminedichloro-platinum (II) stimuli. Our phylogenetic and functional data not only provide a better understanding of the evolution of lamprey PDCD genes but also reveal the conservation of PDCD genes in apoptosis. Overall, our results provide a novel perspective on lamprey immune regulation mediated by the PDCD family.

## Introduction

More than 50 years ago, the phenomenon of cell death during insect development, which is referred to as programmed cell death (PDCD), was observed. Subsequently, increasing analogous cell death phenomena were identified and renamed as apoptosis (1, 2). By the 1990s, many apoptosis regulators were identified, and apoptotic cells have been found to play an important role in the immune system. Currently, there is evidence that the adaptive immune system has developed a unique manner of protecting itself from pathogens. In this process, programmed cell death plays a crucial role (3). Programmed cell death, which is a cellular biological phenomenon, participates in removing foreign substances, and abnormal cells. It also plays an important role in the evolution of organisms, the stability of the internal environment and the development of systems. This abnormal regulatory process is closely related to immune diseases, developmental disorders, and other human diseases (4). Therefore, programmed cell death not only is a distinct type of cell death but also has significant biological significance and complex molecular biological mechanisms. Currently, there are two kinds of genes that regulate programmed cell death, and one of these types of genes inhibits cell death. The other type of gene initiates and promotes cell death. These genes interact to regulate normal cell death and are highly conserved between different species and genera. PDCD gene family members are widely expressed in normal adult tissues, and their expression in cancer tissues and cell lines is upregulated or downregulated depending on the type of tumor tissue or cell line. Currently, there are no reports on whether there is a definitive link between the genes in the PDCD gene family. However, it has been reported that there are significant changes in the genes of the PDCD gene family according to transcriptome data of the overexpression of certain apoptosis genes and after drug treatment (5). Although the genes of this family play a crucial role in the process of apoptosis, there are some differences in the functions of different cell lines. If we elucidate and analyze the functions of these regulatory genes and develop drugs that promote or inhibit the functions of these genes, then we can mitigate the morbidity of diseases such as cancer. To date, 12 genes of the PDCD gene family have been identified in the human genome according to an NCBI query, but currently, there are 7 main genes that are used for mechanistic apoptosis regulation research, which include PDCD1, PDCD2, PDCD4, PDCD5, PDCD6, PDCD7, and PDCD10.

PDCD1 plays essential roles in regulating the response of the immune system and promoting self-tolerance by suppressing T cell inflammatory activity (6). PDCD2 plays an important role during embryonic development and stem cell differentiation (7–9). PDCD4 plays significant roles in tumor promotion, progression, differentiation, and metastasis (10–12). PDCD5 was first identified as a gene that is upregulated in TF-1 cells undergoing apoptosis (13). PDCD5 plays a role in regulating the HDAC3-Akt interaction in the context of endothelial function (14). PDCD6 is associated with cell proliferation and death (15, 16). The PDCD7 protein is involved in glucocorticoid and staphylococcal-induced apoptotic pathways and in ceramide-mediated signaling (17). The PDCD10 gene was initially cloned utilizing a human myeloid cell line by depriving granulocyte macrophage colony-stimulating factor (GM-CSF) (18). Previous studies have suggested that the PDCD10 protein may play an important role in protein synthesis, apoptosis, cell proliferation, and cancer (18–20).

Although the sequences of the PDCD family have been identified in various animals, the characterization and expression profiles of the PDCD family are extremely insufficient in primitive vertebrates. To gain insight into the evolution of PDCD family molecules, we conducted an analysis of multiple PDCD gene family in vertebrates, including the analysis of gene orthologs, conserved synteny, functional domains, and phylogenetic trees. The distribution of the lamprey PDCD family and the immune response against pathogens were also demonstrated. In addition, we investigated the roles of L-PDCD2, L-PDCD5, and L-PDCD10 in apoptosis using L-PDCD2-, L-PDCD5-, and L-PDCD10-overexpressing H293T cell lines. We further investigated whether these changes in cellular processes caused by the overexpression of L-PDCD5 are associated with the BAX and TP53 signaling pathways. Thus, further functional analysis of the lamprey PDCD family could clarify whether PDCD-mediated extrinsic apoptosis and the PDCD-dependent innate immune system are completely developed in lampreys, which are the most primitive vertebrates.

## Materials and Methods

### Sequence Analysis, Multiple Alignments, and Phylogenetic Analysis

The amino acid sequences of the lamprey PDCD family members from a three-generation sequencing library and the amino acid sequences of the corresponding PDCD family genes in other species from NCBI were input into Clustal X 1.83 software for multiple sequence alignment, and a phylogenetic tree was then constructed by the neighbor joining (NJ) method using MEGA 6.0 software and 1,000 booby-trapped replicates. Functional domain analysis of the L-PDCD family genes was conducted using the SMART website (http://smart.embl-heidelberg.de/), and the 3D structure of the L-PDCD family genes was predicted by the online software SWISS-MODEL (http://swissmodel.expasy.org/interactive). Motif analysis was performed by MEME software with the following parameters: zero repetitions per sequence or one repetition per sequence, a motif size between 6 and 50 aa, and a maximum of 15 motifs (21).

### Synteny Analysis of the PDCD Family Genes

For the synteny analysis, the chromosomal locations and lengths and the directionality of neighboring genes upstream (5′) or downstream (3′) of the PDCD family genes were extracted from Ensembl and NCBI genomic databases. To facilitate cross-species comparisons, the neighboring genes of the PDCD family genes from the human genome were used as reference genomic regions. Similarly, the neighboring genes of the PDCD family genes in *Lampetra morii* Berg were used as reference genomic regions for cross-species comparison in fish. For instances of uncertain identity (e.g., genes annotated with numerical identifiers), similarity searches were conducted to establish possible homology relationships between genes.

### Quantitative Real-Time PCR (Q-PCR)

Adult *Lampetra morii* Berg (length: 20–25 cm, weight: 18–23 g) were captured from the YaLu River near DanDong, China, and were housed in glass tanks with filtered fresh water at Liaoning Normal University. Water at a temperature 4°C was replaced on alternating days, and after some time, 54 lampreys were equally assigned to 18 groups, separately immunized with *vibrio anguillarum* (1 × 10^7^ cells/fish), *staphylococcus aureus* (1 × 10^7^ cells/fish), and Poly I:C (100 μg/fish) by intraperitoneal injection, and collected at 2, 8, 24, 48, and 72 h post infection. Animals injected with PBS were used as controls. The gene expression levels of L-PDCD2, L-PDCD4, L-PDCD5, L-PDCD6, and L-PDCD10 in the gill, intestine, liver, kidney, heart, supraneural body, and leukocytes of the lampreys infected with different pathogens were analyzed by quantitative real-time PCR (Q-PCR). Total RNA was extracted from each lamprey tissue sample using TRIzol (Invitrogen, USA), and reverse transcription (No-RT) was performed with a PrimeScript RT-PCR kit (TaKaRa, China) (22). cDNA was used as a template to determine the mRNA expression of the L-PDCD family genes. The sequences of the primers used for Q-PCR are shown in Table 1. Q-PCR was carried out in triplicate using a TaKaRa PCR Thermal Cycler Dice Real Time System, and L-GAPDH was used as an internal control.

**Table 1:** The sequences of primers used for quantitative real time-polymerase chain reaction.

**Species**	**Name**	**Forward primer (5^**′**^/3^**′**^)**	**Reverse primer (5^**′**^/3^**′**^)**
Lamprey	L-PDCD2	GCTCTTTCGAGTTCCAGGTGAT	GTGCACAAAATCCTGCTTCCA
	L-PDCD4	TTCCCGGGCAAATTTACGA	TTGGTGTCTCCGTGCTCAAAG
	L-PDCD5	CAGGGAGCTGGCCATATCTC	GGGACACTGAAATGCGAAACA
	L-PDCD6	GCAGCTCATGATGGGAATGTT	AGCTCCTGCTTGTCGATGAAG
	L-PDCD10	AACCGAAGGGCCCTAGAGAA	CAGGATGGCGTTGGTCTGAT
	L-GAPDH	AGGTGAAGGTCGGAGTCAACGGA	TCAAAGGTGGAGGAGTGGGTGTC
Human	caspase-3	CATGGAAGCGAATCAATGGACT	CTGTACCAGACCGAGATGTCA
	TP53	CAGCACATGACGGAGGTTGT	TCATCCAAATACTCCACACGC
	Bcl2	GGTGGGGTCATGTGTGTGG	CGGTTCAGGTACTCAGTCATCC
	Bax	GTTTGCCCTCGGATCTCTGG	GCTTCCAACAGCGTAAATCCAA
	GAPDH	ATGTTCGTCATGGGTGTGAAC	GCATGGACTGTGGTCATGAGT

The mRNA expression of apoptotic molecules (BAX, BCL-2, TP53, and Caspase 3) was examined in H293T cells transfected with pEGFP-N1 or pEGFP-N1-PDCD5 for 24 h with or without 10^3^ μmol/L cis-diaminedichloro-platinum(II) (CDDP). The PCR primers for Q-PCR are shown in Table 1, and the method was identical to the above mentioned method.

### Purification of the L-PDCD5 Protein and Preparation of an anti-L-PDCD5 Polyclonal Antibody

A plasmid with PDCD5 was used as a template to amplify the target fragment with *Bam*H I and *Hin*d III restriction sites, and the fragment was subcloned into the pCold I vector (TaKaRa, Japan) with a histidine (His) tag. The pCold I-L-PDCD5 plasmid was transfected into BL21 cells, and rL-PDCD5 protein expression was induced with 0.1 mM IPTG (Sangon Biotech, China) for 24 h at 16°C. Subsequently, the recombinant L-PDCD5 protein was purified using a Ni-NTA His-Bind resin column, and the rL-PDCD5 protein concentration was measured using a bicinchoninic acid (BCA) protein assay kit (Beyotime, China). The purified rL-PDCD5 protein was analyzed by 15% SDS-PAGE and used as an antigen. Then, adult New Zealand white rabbits were periodically immunized six times, and the antibody was purified using a CNBr-activated Sepharose 4B column (GE Healthcare, Germany) according to the manufacturer's instructions. The coupling solution containing the ligand of the rL-PDCD5 protein was mixed with prepared CNBr-activated Sepharose 4B medium suspension in a stoppered vessel for 1 h. Then, rabbit serum was collected and subjected to the coupling solution equilibrated with binding buffer (0.5 M NaCl, 100 mM Tris-HCl, and 0.1 M acetic acid/sodium). The antibody titer was determined by an enzyme-linked immunosorbent assay (ELISA) and western blotting.

### Mass Spectrometry and Protein Identification

The molecular mass and purity of the protein were analyzed by 15% SDS-PAGE, and protein bands were observed by staining with 0.25% Coomassie Brilliant Blue R-250. Strips were cut with a clean blade and placed in a decolorizing solution containing 35% acetonitrile and 65% ammonium bicarbonate, and in-gel tryptic digestion was performed according to the manufacturer's instructions. LC/MS/MS of tryptic peptides was performed by MALDI-TOF mass spectrometry using a Bruker Ultraflex mass spectrometer. The obtained PMF-lift profiles were used to search for peptide matches using ProFound, which was run locally against our expression sequence tags (ESTs) lamprey cDNA library database.

### High-Content Screening

MCF-7 cells were plated in 96-well plates at a density of 10^5^ cells/well and treated with Alexa 488-labeled rL-PDCD5 (Molecular Probes, Invitrogen Corporation, Carlsbad, CA) for 15 min and 2, 5, and 24 h. The cells were washed twice with phosphate-buffered saline (PBS) and stained with Hoechst (Sigma) for 20 min for the observation of cell nuclei. The samples were analyzed on a high-content screening system (PerkinElmer, USA).

### Confocal Microscopy

H293T and HeLa cells were transfected with pEGFP-N1, pEGFP-N1-PDCD2, pEGFP-N1-PDCD5, and pEGFP-N1-PDCD10 for 24 h with Lipofectamine® 3,000 (Thermo Fisher Scientific) according to the manufacturer's instructions in 8-well chamber slides (Nunc, Thermo Fisher Scientific). Immunofluorescence was observed and imaged with a Zeiss LSM 780 inverted microscope (Carl Zeiss, Inc.,) and analyzed using Zeiss ZEN LE software.

Lamprey blood was taken out from the tail and leukocyte of lamprey are obtained by Ficoll-Paque gradient centrifugation of the blood with lymphocyte separating solution. Then, leukocytes were added to tubes and fixed for 10 min in 70% ethanol in PBS at room temperature. Then, the cells were permeabilized with 0.1% Triton X-100 for 30 min, blocked with 10% bovine serum albumin (BSA) for 30 min, and incubated with the rabbit anti-L-PDCD5 antibody (5.0 μg/mL) in PBS for 1 h at room temperature. After the cells were washed three times with PBS, the cells were incubated with Alexa Fluor 488 goat anti-rabbit IgG (5.0 μg/mL) for 45 min at room temperature in the dark. The leukocytes were stained with DAPI (1,000-fold dilution) followed by two washes with PBS. Immunofluorescence was observed and imaged with a Zeiss LSM 780 inverted microscope (Carl Zeiss, Inc.,) and analyzed using a FACSAria flow cytometer (BD Biosciences).

### Live Cell Imaging

HeLa cells were transfected with pEGFP-N1-PDCD5 for 24 h in 8-well chamber slides (Nunc, Thermo Fisher Scientific). For HeLa cells, we acquired 6 z-sections at a step size of 0.75 μm in the Hoechst 33342, GFP and bright-field channels. Image acquisition was conducted using a DeltaVision imaging system (GE Healthcare). Time-lapse images of the cells were acquired every 10 min for 24 or 48 h.

### Western Blotting Analysis

Western blotting was used to analyze the protein expression levels of L-PDCD5 in cell lysates. 21 lampreys were equally assigned to 7 groups, separately immunized by intraperitoneal injection with *vibrio anguillarum* (1 × 10^7^ cells/fish) and grass carp reovirus (GCRV) (2 × 10^8^ pfu/fish), and collected at 12, 24, and 48 h post infection. Animals injected with PBS were used as controls. After measuring the protein concentration, protein samples were separated by 15% SDS-polyacrylamide gel electrophoresis (SDS-PAGE) and transferred onto polyvinylidene difluoride (PVDF) membranes. The membranes were blocked with 5% skim milk for 2 h and subsequently probed overnight at 4°C with primary antibodies against the rabbit anti-rL-PDCD5 antibody followed by incubation with HRP-conjugated goat anti-rabbit IgG (5,000-fold dilution). The membrane was developed using an ECL substrate (Beyotime, China). Relative quantification of the bands was performed using ImageJ software.

The protein expression of apoptotic molecules (BAX, BCL-2, TP53, and Caspase 3) was examined in H293T cells transfected with pEGFP-N1 or pEGFP-N1-PDCD5 for 24 h with or without CDDP (10^3^ μmol/L) using western blotting. We used primary antibodies against GFP (23), Caspase 3 (24), TP53 (25), BCL-2 associated X protein (BAX) (26), B-cell lymphoma 2 (BCL-2) (27), and β-actin (used as a loading control) (28) at dilutions of 1:800, 1:1,000, 1:1,000, 1:1,000, 1:1,500, and 1:1,000, respectively (ABclonal, USA). Secondary antibodies were used as described above.

### Apoptosis Assay

For the Annexin V-FITC/PI apoptosis assay, MCF-7 and H293T cells were exposed to the rL-PDCD5 (0.3 μg/μL) protein alone for 24 h or in combination with CDDP (20 μmol/L) for 6 h at 37°C. The cells were then collected, washed with PBS, and stained with Annexin V-FITC/PI (Tianjin Sungene Biotech Co., Ltd., China) according to the manufacturer's instructions. The cells were then analyzed by a FACSCalibur flow cytometer (Becton Dickinson, CA). The flow cytometer was set at 488 and 560 nm (excitation wavelengths) to detect green and red fluorescence, respectively. For the Alexa Flour® 555 Annexin V conjugate apoptosis assay, H293T-pEGFP-N1, H293T-pEGFP-N1-PDCD2, H293T-pEGFP-N1-PDCD5, and H293T-pEGFP-N1-PDCD10 cells were treated with or without CDDP (10^3^ μmol/L) for 6 h. Apoptosis was detected with Annexin V conjugate staining (Invitrogen, USA) according to the manufacturer's instructions, and the samples were immediately analyzed by flow cytometric analysis. The flow cytometer was set at 560 nm (excitation wavelength) to detect red fluorescence, and analyses were performed by using Modfit software. Three independent experiments were performed.

### Cell Proliferation and Cytotoxicity Assay

Cell proliferation and cytotoxicity were analyzed with a cell counting kit-8 (CCK-8) assay. H293T cells were incubated in a 96-well plate (3 × 10^4^ cells/well), transfected with the recombinant plasmid for 24 h and then either treated with or without CDDP (10^3^ μmol/L) for 6 h. Subsequently, 10 μL of CCK-8 solution was added to each well, and the cells were further incubated at 37°C for 4 h. The absorbance was measured at a wavelength of 450 nm, and the reference absorbance was obtained at a wavelength of 630 nm with a microplate reader (Thermo, Waltham, MA, USA).

### Statistical Analysis

All data are presented as the mean ± SD based on separate experiments. Differences between treatment groups were determined by Student's *t*-test. *P* < 0.05 was set as the threshold for statistical significance (^*^P < 0.05, ^**^P < 0.01, ^#^*P* < 0.05, and ^##^*P* < 0.01).

## Results

### Identification and Sequence Analysis of PDCD Family Genes

**Figure 1:**
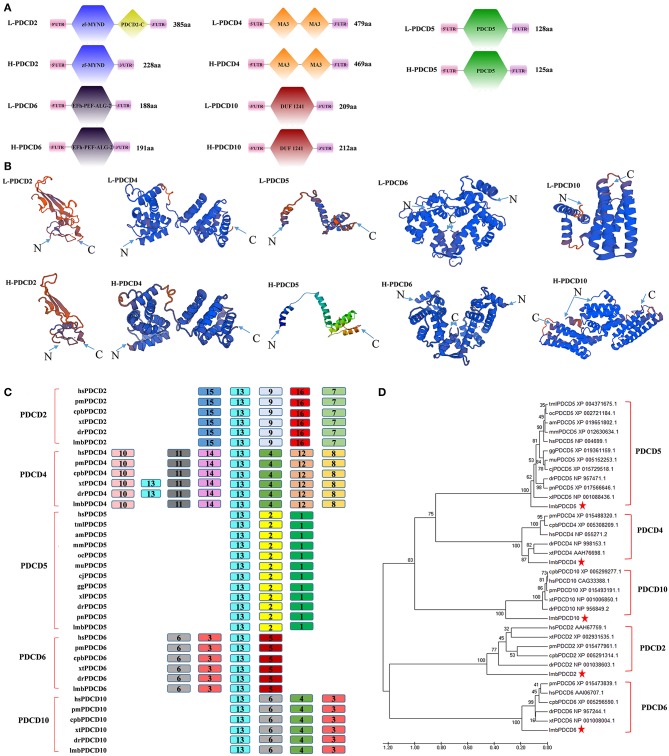
The identification and sequence analysis of PDCD family genes. **(A)** A comparison of the domains between PDCD2, PDCD4, PDCD5, PDCD6, and PDCD10. **(B)** Tertiary structure prediction of PDCD2, PDCD4, PDCD5, PDCD6, and PDCD10 from humans and lampreys. **(C)** A motif composition of the PDCD family proteins. The motifs, which are numbered 1-16, are shown in different colored boxes. **(D)** The phylogenetic tree for the PDCD family based on the NJ method. An NJ tree was constructed using the amino acid sequences of the PDCD proteins. The numbers at the nodes indicate the bootstrap confidence values derived from 1000 replications. hs, Homo sapiens; lmb, Lampetra morii berg; pm, Parus major; cpb, Chrysemys picta bellii; xt, Xenopus tropicalis; dr, Danio rerio; tml, Trichechus manatus latirostris; oc, Oryctolagus cuniculus; am, Ailuropoda melanoleuca; mm, Microcebus murinus; gg, Gavialis gangeticus; mu, Melopsittacus undulatus; cj, Coturnix japonica; pn, Pygocentrus nattereri; and xl, Xenopus laevis. The bar (0.020) indicates the genetic distance.

To reveal when these apoptotic molecules originated and how their function in promoting apoptotic signals evolved, we began with a search for PDCD-related sequences in lampreys. The lamprey genome contains at least five PDCD genes, which include PDCD2, PDCD4, PDCD5, PDCD6, and PDCD10. As shown in Table 2, we identified and analyzed multiple sequence alignments of the PDCD gene family. Based on the sequence, alignment and 3D structure analysis, there were no similarities in the structural domains between the PDCD family genes. Indeed, sequence analysis also revealed that the lamprey PDCD genes are highly homologous with the human PDCD family (Figures 1A,B). To investigate the conservation of the PDCD genes, motifs were analyzed between the PDCD protein families, and 16 distinct motifs were identified by the MEME motif search tool (Figure 1C and Table 3). Motif 13, which is a representative domain, was identified in all lamprey PDCD protein families. Lamprey PDCD2 proteins contain 5 motifs (motifs 7, 9, 13, 15, and 16). PDCD4 proteins contain 7 motifs (motifs 4, 8, 10, 11, 12, 13, and 14). PDCD5 proteins contain 3 motifs (motifs 1, 2, and 13). Interestingly, most of the specific motifs were observed in PDCD proteins. For example, motifs 3, 6, and 13 were identified in all members of the PDCD6 and PDCD10 proteins, whereas motif 5 was only observed in the PDCD6 family. Therefore, individual motifs may play important roles in biological activity. To clarify the evolutionary process of the lamprey PDCD families, phylogenetic trees based on the NJ method from vertebrate PDCD family genes were constructed and supported our speculation (Figure 1D). Moreover, the phylogenetic trees confirmed that the lamprey PDCD protein is located in different clusters and is found in the outer group of vertebrates, indicating that the lamprey PDCD protein is unique and conserved. In summary, the results of the gene structure and conserved motif analyses support the results of the phylogenetic analysis, indicating that the evolution of each PDCD gene is conserved in all species.

**Table 2:** Identification and sequence analysis of lamprey PDCD gene families.

**Gene**	**Open reading frame (ORF)**	**Amino acids**	**Molecular mass (kDa)**	**Isoelectric point**	**Formula**	**Sequence identity (%)**
L-PDCD2	1158bp	385	42.60148	5.13	C_1848_H_2834_N_540_O_578_S_23_	51.5
L-PDCD4	1440bp	479	52.99711	5.54	C_2318_H_3711_N_679_O_710_S_17_	60.9
L-PDCD5	387bp	128	14.53532	5.56	C_1526_H_2549_N_505_O_634_S_168_	75
L-PDCD6	567bp	188	21.78151	5.33	C_974_H_1451_N_267_O_283_S_11_	66.3
L-PDCD10	630bp	209	23.52393	6.85	C_1039_H_1687_N_289_O_319_S_6_	59.2

**Table 3:** The conserved motifs discovered among the amino acid sequences of the PDCD gene families (without the signal peptides) from vertebrates and invertebrates using the MEME system.

**Motif**	**Width**	**Best possible match**
1	41	NYLIQMARYGQLPGKVSEQGLIEILEKVSQQTEKKTTVKFN
2	50	HGDPGDAAQQEAKHREAEMRNSILAQVLDQSARARLSNLALVKPDKAKAV
3	41	YVKSFSDMFKTYFKDGKNFNVFTSVWKYIHQWNNIFRTYKT
4	41	MRAGKQILDNIWGEINGQVRFKHTIKEIAMAIKEYLDSGNN
5	50	DRQGRGQVAFDDFIQCCIVLQRLTDVFRRYDTDQDGWIQVSYEQYLSMVF
6	41	LPDQQFLWNVFQRVDKDNPGVISDTIMQQALSNGTWTPFNP
7	50	EKDIPDCSCGAKRIFEFQVMPQLLNHLKVDSLGESIDWGTLVVYTCAESC
8	50	WKSGVITLDQMNRGYERVYGEIPDINLDVPHAYSVLERFVEECFQAGIIT
9	45	LKCGAHLCRVCGCLGPKACSRCHKAHYCCKEHQTMDWKLGHKHSC
10	50	KGKLLDRRSRSGKGRGLPKKGGAGGKGVWGTPGQVYDVEEVDIKDPNYDD
11	50	EYFEHGDTNEVAEMLKDLNLGHMKYGVPVLAVSLALEGKASHREMTSKLL
12	41	ERCLRELEVPHFHHELVYEAIVMVLESTGEATFKMMVDLLK
13	15	CMFLYAFCYQVYAEL
14	50	LCGTVLSPTDMEKAFDRMLKELPELILDTPRAPQMVGQFIARAVGDHILP
15	41	GFPEPAPAWRLRSAQFPSKVGGRPAWLGEAGLPGPAALQCG
16	35	LEAMAKHETREDKIFQKFKKRIAAEPEQILRYCRG

### A Comparison of Syntenic Genomic Regions Containing L-PDCD Genes

**Figure 2:**
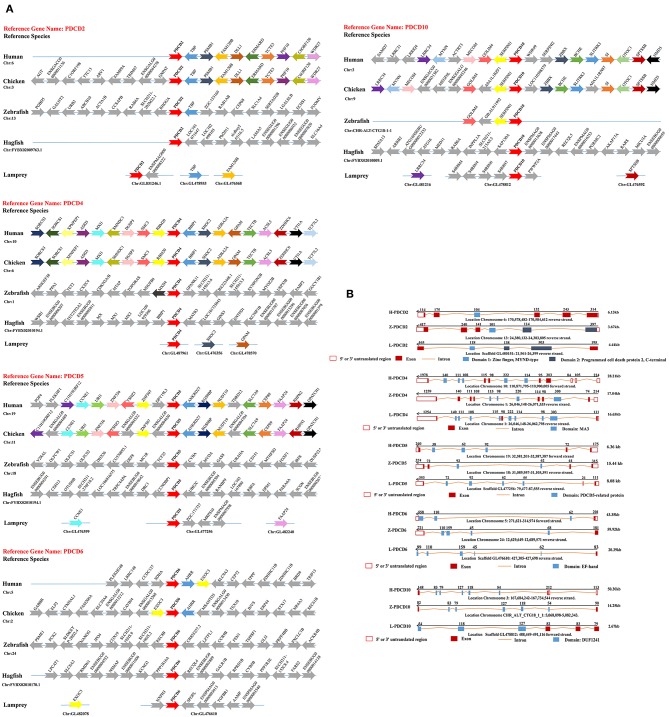
Syntenic relationships and gene structure in the PDCD family. **(A)** The conservation of genes neighboring PDCD family genes. The genes (10 kb) are indicated by arrowheads. The arrows and arrowheads pointing in opposite directions indicate genes located on opposite strands. Chr denotes the chromosome. **(B)** A representation of the available sequence data for the PDCD family genes. Noncoding exons are designated with open boxes, and coding exons are indicated by colored boxes. The transcription start site is indicated by an arrow, and the sizes of the 5' introns are labeled. PDCD exons that encode the domain are labeled.

The investigation of genes in lampreys and hagfish provides a unique perspective on the evolutionary genetic foundations of the evolution of vertebrate genomes. Genomic synteny and comparisons are useful not only for establishing gene homology relationships but also for providing clues regarding the mechanistic origin of genes (29). To better understand the evolution of the PDCD gene family during vertebrate radiation, the neighboring gene environment of lamprey PDCD was compared between fish and mammals. In humans, the PDCD2, PDCD4, PDCD5, PDCD6, and PDCD10 genes are mapped to chromosomes 6, 10, 19, 5, and 3, respectively. A chromosome region with a similar gene repertoire to that flanking the human PDCD gene family was found in chickens. In the lamprey genome, GL831246.1 surrounding L-PDCD2, GL487961 surrounding L-PDCD4, GL477256 surrounding L-PDCD5, GL476610 surrounding L-PDCD6, and GL478812 surrounding L-PDCD10 were not found in the neighborhood of gnathostome PDCD family genes. Strong syntenic relationships between PDCD gene family orthologs were easily detected in the human and chicken genome sequences that we examined; however, in the zebrafish genome, syntenic relationships were not easily detected due to differential gene losses after TGD (Figure 2A). In the lamprey genome, neighboring genes, although detectable, could not be examined for syntenic relationships with the mammalian PDCD gene family (Figure 2A). The neighborhood of the lamprey PDCD gene family was not found to share a gene linkage with any of the vertebrate chromosomes/scaffolds containing PDCD genes, and its location may be the result of gene duplication and subsequent translocation. Moreover, compared with human PDCD genes, L-PDCD genes have substantial differences in exons and introns (Figure 2B).

### The Expression Pattern of PDCD Genes in Lamprey Tissues and the Immune Response to Various Pathogenic Challenges

**Figure 3:**
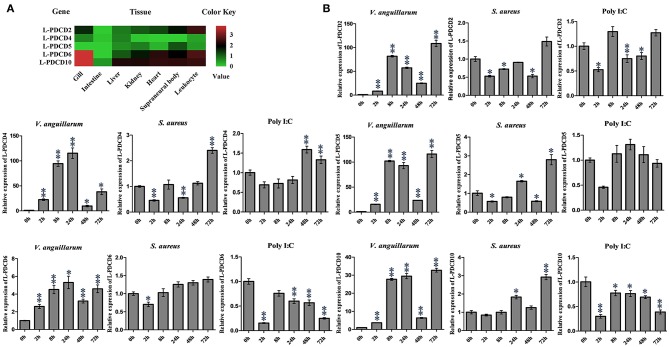
The expression profiles of lamprey PDCD family genes. **(A)** RT-PCR analysis of the L-PDCD family genes in lampreys characterizing L-PDCD mRNA expression in adult tissues. The mRNA expression levels of L-PDCD family genes are expressed as a ratio relative to the GAPDH mRNA expression levels. The expression profile for lamprey PDCD family genes are shown by using a heat map. The green and red colors indicate low to high expression levels. **(B)** Q-PCR analysis of the expression patterns of L-PDCD2, L-PDCD4, L-PDCD5, L-PDCD6, and L-PDCD10 at 0, 2, 8, 24, 48, and 72 h after the challenges with Gram-negative *V. anguillarum* (1 × 10^7^ cells/fish), Gram-positive *S. aureus* (1 × 10^7^ cells/fish), and the viral mimic pathogen Poly I:C (100 μg/fish) in the leukocytes of lampreys. All of the data are presented as the means ± SDs based on three independent cDNA samples with three replicates per sample. The asterisks indicate significant differences (*n* = 3, ^**^P < 0.01 and ^*^P < 0.05) compared to the control.

The expression of L-PDCD2, L-PDCD4, L-PDCD5, L-PDCD6, and L-PDCD10 genes in various lamprey tissues was analyzed by Q-PCR and shown as a heatmap (Figure 3A). In all instances, the GAPDH gene was amplified successfully, indicating the validity of the cDNA template. The transcripts of all characterized L-PDCD family genes, especially L-PDCD4, and L-PDCD5, were less abundant in the lamprey tissues. However, the level of L-PDCD10 expression was greater than that of other L-PDCD family genes in most of the tissues. A high level of L-PDCD2 gene expression was found to be mainly distributed in leukocytes, and the expression level of L-PDCD6 and L-PDCD10 was higher in the gills.

Information regarding the specific roles of PDCD family genes in the immune responses of lamprey currently remains limited. Furthermore, to facilitate an understanding and provide important information regarding the initial immune responses of lampreys and expression changes in the PDCD genes, the expression changes in the PDCD genes in lampreys that were experimentally infected with various pathogens were investigated according to the pathogens used. The results revealed that the overall trend of L-PDCD2, L-PDCD5, L-PDCD6, and L-PDCD10 gene transcription levels were reduced or normal after the mimic virus pathogen Poly I:C attacked the leukocytes of the lampreys (Figure 3B), except for the upregulation of L-PDCD4 expression. In addition, the results also revealed the upregulation of the expression of all L-PDCD genes relative to the control group due to infections with Gram-negative *V. anguillarum* after 2 h in leukocytes. However, upregulation of L-PDCD gene expression was delayed until 72 h after the lamprey leukocytes were infected with Gram-positive *S. aureus*.

### The Roles of L-PDCD5 in the Lamprey Immune Responses

**Figure 4:**
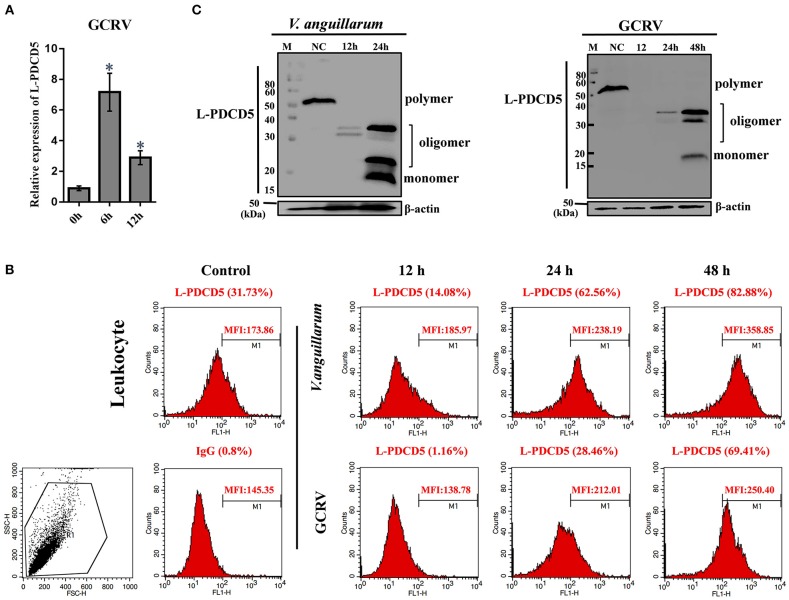
The functions of L-PDCD5 in the immune responses of lampreys. **(A)** Q-PCR analysis of the expression patterns of L-PDCD5 in leukocytes at 0, 6, and 12 h after lampreys were challenged with GCRV. All of the data are presented as the means ± SDs based on three independent cDNA samples with three replicates per sample. The asterisks indicate significant differences (*n* = 3 and ^*^P < 0.05) compared to the control. **(B)** Immunofluorescence analysis of PDCD5 surface expression in leukocytes at 0, 12, 24, and 48 h after lampreys were challenged with or without *V. anguillarum* and GCRV treatment were bound to the rabbit anti-rL-PDCD5 antibody and incubated with FITC-conjugated anti-rabbit IgG secondary antibodies followed by FACS analysis. Numbers above bracketed lines indicate percent PDCD5 positive cells mean fluorescence intensity. Percentages indicate the number of PDCD5 positive cells. **(C)** Western blotting analysis of the L-PDCD5 expression in leukocytes at different periods after the challenges with *V. anguillarum* and GCRV infection using the rabbit anti-rL-PDCD5 antibody. β-actin was used as a loading control. All experiments were repeated at least three times with similar results.

To determine the role of L-PDCD5 in immune response. The expression patterns of L-PDCD5 in the leukocytes were upregulated after the lampreys were challenged with Gram-negative *V. anguillarum*, Gram-positive *S. aureus*, and GCRV; however, there was no obvious change in the Poly I:C treatment group. Therefore, lampreys were treated with GCRV infection instead of Poly I:C stimulation. The Q-PCR results revealed that the transcription level of the L-PDCD5 gene was enhanced in the leukocytes of the lampreys after the challenge with GCRV, as shown in Figures 3B, 4A.

To further evaluate the expression of the L-PDCD5 protein in leukocytes, recombinant L-PDCD5 was expressed and purified (Figures S1A,B) and L-PDCD5 antibodies were prepared (Figures S1C,D). Lamprey leukocytes with or without pathogen treatment were incubated with anti-rL-PDCD5 protein polyclonal antibodies and analyzed by flow cytometry. FACS analysis revealed that L-PDCD5 was expressed in approximately 31.73% of the leukocytes without the pathogen treatment. These results indicated that the number of L-PDCD5-expressing cells first decreased and then increased with increasing stimulation time. Preimmunized rabbit IgG was used as a negative control (Figure 4B). Then, we investigated the protein expression level of L-PDCD5 in lamprey leukocytes through western blotting. Furthermore, we observed typical L-PDCD5 polymers in lamprey leukocytes. Interestingly, L-PDCD5 polymers disappeared or decreased, but oligomers and monomers were observed in leukocytes against *V. anguillarum* and GCRV (Figure 4C). Furthermore, LC/MS/MS analysis of tryptic-digested peptides of the oligomer and monomer band proteins identified 11 unique peptides with >95% probability (Table 4). When these newly identified peptide sequences were explored in the lamprey protein databases were searched, these sequences showed significant similarity (score: 226.41, rank: 1, and 43% sequence coverage) and were termed PDCD5. Overall, these results suggest that L-PDCD5 in monomers most likely participates in innate immune responses.

**Table 4:** LC/MS/MS analysis of tryptic-digested peptidesn of PDCD5.

**m/z mes**	**Score**	**Sequence**
481.7426	54.8	R.MSELQAQR.G
489.7400	57.0	R.MSELQAQR.G
573.7634	83.8	R.GGAGGTDQEAQR.E
599.3686	94.4	R.LSNLALVKPDK.A
653.3668	73.8	K.SVENYLIQLAR.Y
381.7131	29.8	R.YGQLPGK.V
610.3230	35.0	R.YGQLPGKVDDK.G
686.3940	58.1	K.VDDKGLIEILEK.V
457.7843	22.6	K.GLIEILEK.V
680.2701	23.1	R.KVMDSDEEDEY
688.2680	32.3	R.KVMDSDEEDEY

### Cellular Uptake of the Exogenous rL-PDCD5 Protein Induces Apoptosis

**Figure 5:**
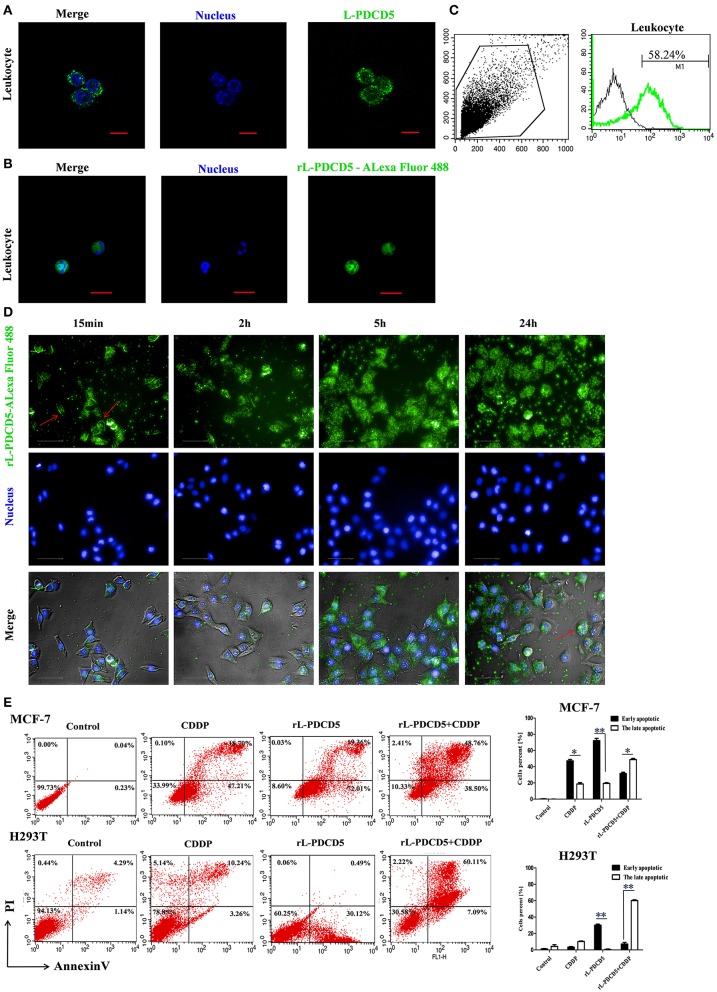
The effect of rL-PDCD5 on morphological changes and apoptosis of leukocytes, MCF-7 cells, and H293T cells. **(A)** Immunofluorescence detection of L-PDCD5 in leukocytes. The primary antibody was the rabbit anti-rL-PDCD5 antibody (5.0 μg/mL), and the secondary antibody was an Alexa Fluor 488-conjugated goat anti-rabbit IgG antibody (5.0 μg/mL). The cells were observed and imaged with a Zeiss LSM 780 inverted microscope (Carl Zeiss, Inc.,) and analyzed using Zeiss ZEN LE software (60 × magnification). Scale bars: 5 μm. **(B)** Immunofluorescence detection of leukocytes treated with Alexa 488-labeled rL-PDCD5. The leukocytes were incubated in medium containing Alexa 488-labeled rL-PDCD5 (1.0 μg/μL) for 24 h and then incubated with Hoechst 33258 nucleic acid stain for 4 min. **(C)** FACS analysis of the efficiency of Alexa 488-labeled rL-PDCD5 entry into leukocytes. The leukocytes were incubated in medium containing Alexa 488-labeled rL-PDCD5 (1.0 μg/μL) for 24 h and then analyzed by FACS. **(D)** High-content screening analysis of MCF-7 cells treated with Alexa 488-labeled rL-PDCD5. MCF-7 cells were incubated with Alexa 488-labeled rL-PDCD5 (1.0 μg/μL) in a time-dependent manner and then incubated with Hoechst 33258 nucleic acid stain for 4 min. The cells were observed using high-content screening and photographed at the indicated time points (40 × magnification). **(E)** MCF-7 and H293T cells were exposed to the rL-PDCD5 (0.3 μg/μL) protein alone for 24 h or in combination with CDDP (20 μmol/L) for 6 h at 37°C. Apoptosis was analyzed by Annexin V/FITC and PI staining followed by flow cytometry. A histogram shows the statistics of the above mentioned results. The data are presented as the means ± SDs; *n* = 3, ^*^P < 0.05, and ^**^P < 0.01.

Recombinant human PDCD5 protein (rhPDCD5) enters cells through a clathrin-independent endocytic pathway, which involves cell surface heparan sulfate proteoglycans and lipid rafts, and enhances apoptotic activity (30). It has been reported that rhPDCD5 itself cannot directly induce cell apoptosis. Therefore, H-PDCD5 was considered an apoptosis accelerator or enhancer but not an apoptosis inducer (31). Indeed, the immunofluorescence assay showed that the L-PDCD5 protein was mainly localized in the cell membrane and cytoplasm of leukocytes and liver and supraneural body cells, but a small part of the L-PDCD5 protein was dispersed in the nuclei of the lampreys (Figure 5A and Figure S2). In our study, lamprey leukocytes and human cell lines were double-stained with Alexa 488-labeled rL-PDCD5 (1.0 μg/μL) and Hoechst 33258 nucleic acid stain. Interestingly, when the lamprey leukocytes were incubated with Alexa 488-labeled rL-PDCD5 for 24 h, we found that Alexa 488-labeled rL-PDCD5 entered leukocytes (Figure 5B). FACS analysis revealed that Alexa 488-labeled rL-PDCD5 enters leukocytes with an efficiency of ~58% (Figure 5C). Furthermore, the same results indicated that L-PDCD5 significantly moved from the cytoplasm to the nucleus after MCF-7 cells were incubated with Alexa 488-labeled rL-PDCD5 in a time-dependent manner (Figure 5D), suggesting internalization by an endocytic process. H-PDCD5 accelerates apoptosis under the stimulation of various chemotherapeutic drugs (31). However, the effects of recombinant PDCD5 on apoptosis in lampreys remain unknown. Our results of the apoptosis assay by flow cytometry analysis revealed that rL-PDCD5 (0.3 μg/μL) induced cell apoptosis, and a histogram showing the statistics of the above mentioned results is shown in Figure 5E. In addition, when rL-PDCD5 was administered in combination with 20 μmol/L CDDP, a synergistic increase in the number of apoptotic cells was observed. Based on these findings, in contrast to human PDCD5, lamprey PDCD5 efficiently promotes programmed cell death triggered by the cellular uptake of the exogenous rL-PDCD5 protein and induces cell apoptosis.

### The Effect of the Overexpression of L-PDCD2/5/10 on Apoptosis

**Figure 6:**
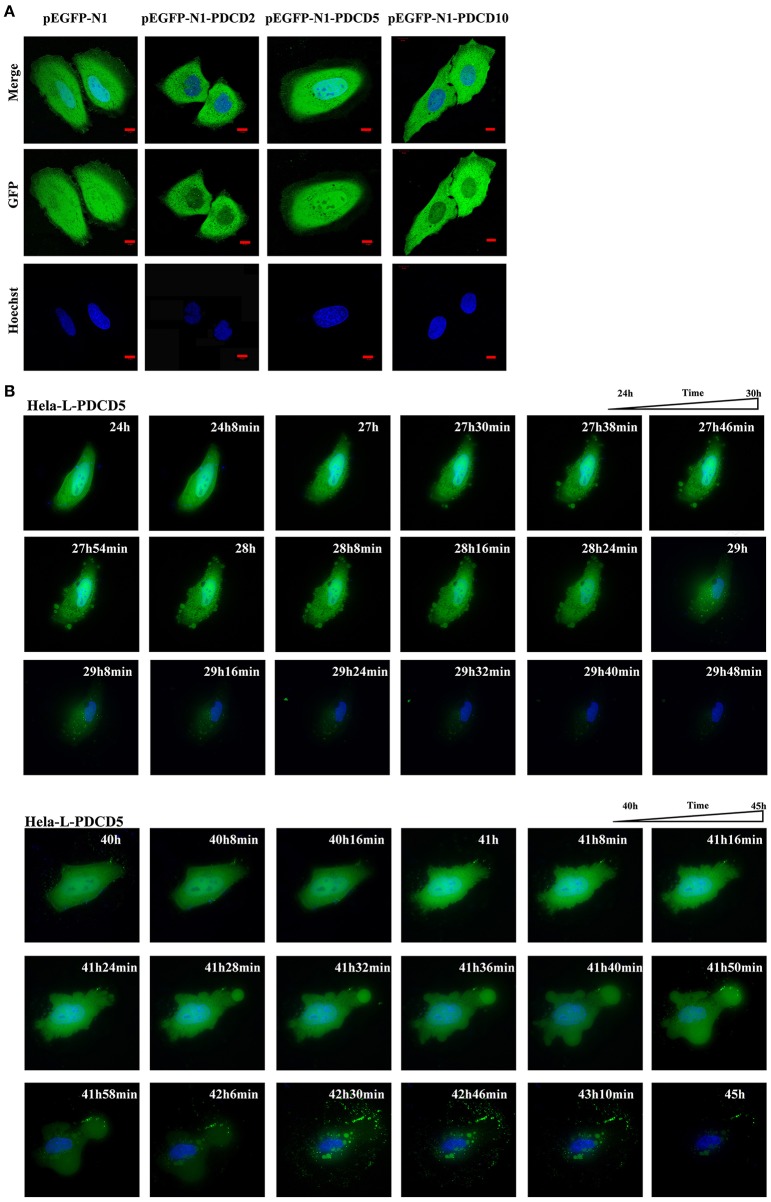
Confocal microscopy and live cell imaging of the overexpression of L-PDCD5 in HeLa cells. **(A)** Confocal microscopy observation of HeLa cells transfected with L-PDCD2, L-PDCD5, and L-PDCD10 for 24 h and then treated either with or without CDDP (10^3^ μmol/L) for 6 h. Hoechst 33342 was used to stain the nucleus. **(B)** Time-lapse imaging of L-PDCD5 overexpression in living cells (also shown in Supplemental Video 1). HeLa cells were transfected with L-PDCD5 for 24–45 h and stained with Hoechst 33342. The bubbling phenomenon was first observed in the cells, and the cells subsequently aggregated with each other until a small aggregate/particle-like structure was formed.

**Figure 7:**
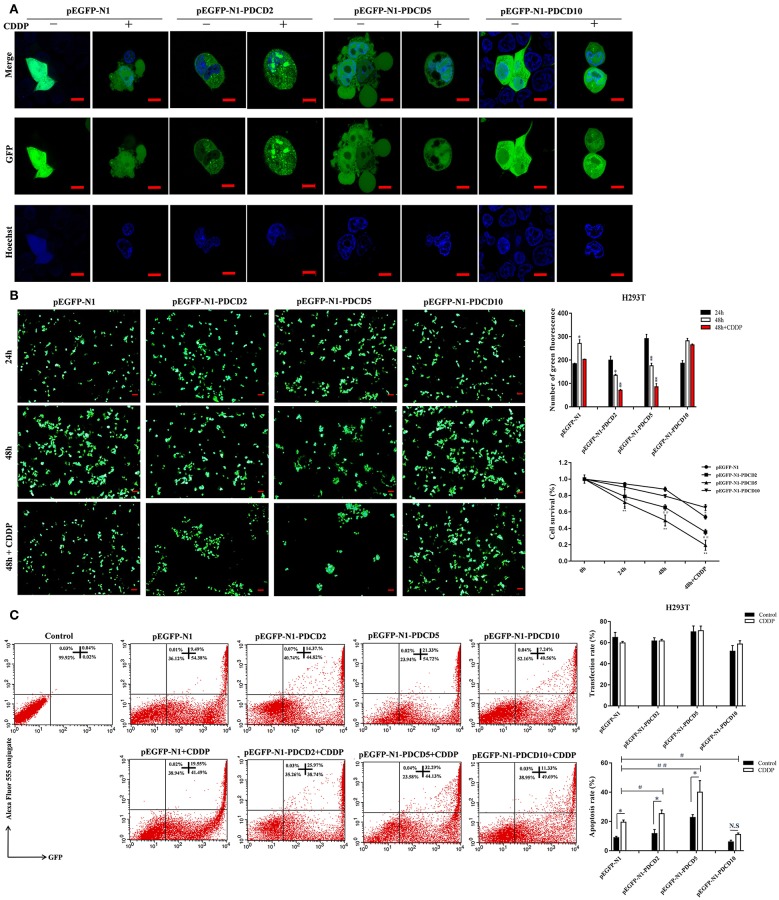
The localization of L-PDCD2, L-PDCD5, and L-PDCD10 and cell morphological changes upon apoptosis were observed in response to DNA damage. **(A)** Confocal microscopy observation H293T cells transfected with L-PDCD2, L-PDCD5, and L-PDCD10 for 24 h and then treated either with or without CDDP (10^3^ μmol/L) for 6 h. Hoechst 33342 was used to stain the nucleus. **(B)** L-PDCD5 enhances the growth inhibition of H293T cells. The left graph shows the transfection efficiency of cells under fluorescence microscopy, while the upper right graph shows the statistics. Furthermore, the lower right graph shows the cell viability of L-PDCD5-overexpressing cells at the indicated time points using a CCK-8 assay. The data are presented as the means ± SDs of the results from three independent experiments (^*^P < 0.05, ^**^P < 0.01, ^#^*P* < 0.05, and ^##^*P* < 0.01). **(C)** H293T cells were transfected with L-PDCD2, L-PDCD5, and L-PDCD10 for 24 h and then treated either with or without CDDP (10^3^ μmol/L) for 6 h. Apoptosis was determined through Annexin V conjugate staining using flow cytometry. L-PDCD2 or L-PDCD5 overexpression increased the apoptotic rate in H293T cells that were either treated with or without CDDP (left side). A histogram showing the statistics of the above mentioned results (right side). All experiments were repeated at least three times with similar results (^*^P < 0.05, ^**^P < 0.01, ^#^*P* < 0.05, and ^##^*P* < 0.01).

To gain a better understanding of the lamprey PDCD family, we investigated the protein localization and function of L-PDCD2, L-PDCD5, and L-PDCD10 on cell fate. Currently, time-lapse microscopy is ideally suited for examining gene expression dynamics and protein localization. After HeLa cells were transfected with L-PDCD2, L-PDCD5, and L-PDCD10 expression vectors, the cells were either treated with or without CDDP (10^3^ μmol/L) for 6 h. Our data showed that L-PDCD2 and L-PDCD10 mainly localized to the cytoplasm, whereas L-PDCD5 was observed in discrete compartments in the cytoplasm and nucleus (Figure 6A). Furthermore, overexpression of the L-PDCD5 gene in HeLa cells was analyzed by a live cell Delta Vision imaging system (Supplemental Video 1). The time-lapse data indicated that HeLa cells underwent obvious morphological changes, including chromatin compaction, membrane blebbing, and cell shrinkage. Finally, L-PDCD5 proteins aggregated with each other until a small aggregate/particle-like structure was formed that was uniformly distributed in the cytoplasm, and this process typically lasted for 4–6 h (Figure 6B). Our results indicate that in contrast to human PDCD5, lamprey PDCD5 plays an important role in inducing apoptosis. Similar to lamprey PDCD5, PDCD2 promotes and accelerates apoptosis in response to CDDP. In contrast, L-PDCD10 was shown to be associated with antiapoptosis by confocal microscopy and immunofluorescence microscopy (Figure 7A). To further verify the extent of apoptosis induced by L-PDCD2/5/10 in H293T cells, we conducted a CCK-8 assay and a single-step staining method for labeling cells with Annexin V-Alexa fluor 555 to detect apoptotic cells by ELISA and flow cytometry. As shown in Figures 7B,C, the overexpression of the L-PDCD2 and L-PDCD5 genes in H293T cells inhibited cell growth and induced apoptosis and notably improved the apoptosis-inducing effects of CDDP. However, compared with the vector alone, the overexpression of the L-PDCD10 gene inhibited the apoptosis-inducing effects of CDDP.

### The Mechanism of L-PDCD5-/L-PDCD10-Induced Apoptosis of H293T Cells

**Figure 8:**
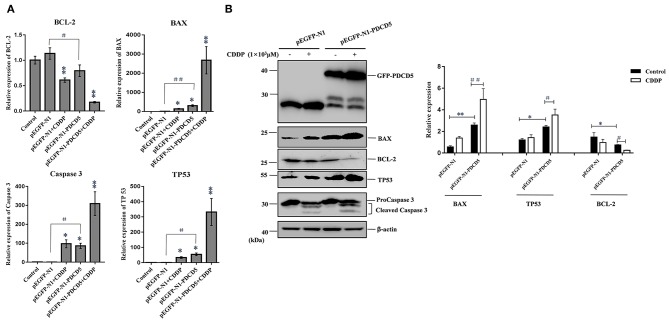
The effect of the exogenous overexpression of L-PDCD5 on apoptosis-related proteins. **(A)** Q-PCR analysis of apoptotic molecule (BAX, BCL-2, TP53, and Caspase 3) expression in H293T cells transfected with L-PDCD5 for 24 h and treated either with or without CDDP (10^3^ μmol/L). Total RNA was quantified by Q-PCR and normalized to GAPDH expression. All experiments were repeated at least three times with similar results (^*^P < 0.05, ^**^P < 0.01, ^#^*P* < 0.05, and ^##^*P* < 0.01). **(B)** Western blotting analysis of BAX, BCL-2, TP53, and Caspase 3 protein expression using specific antibodies. β-actin was used as a loading control (left side). A histogram showing the statistics of the above mentioned results (right side). All experiments were repeated at least three times with similar results (^*^P < 0.05, ^**^P < 0.01, ^#^*P* < 0.05, and ^##^*P* < 0.01).

**Figure 9:**
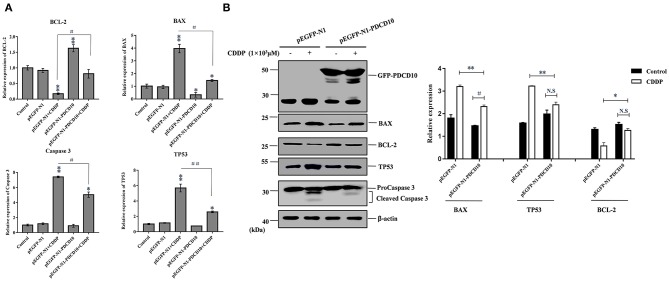
The effect of the exogenous overexpression of L-PDCD10 on apoptosis-related proteins. **(A)** Q-PCR analysis of apoptotic molecule (BAX, BCL-2, TP53, and Caspase 3) expression in H293T cells transfected with L-PDCD10 for 24 h and treated either with or without CDDP (10^3^ μmol/L). Total RNA was quantified by Q-PCR and normalized to GAPDH expression. All experiments were repeated at least three times with similar results (^*^P < 0.05, ^**^P < 0.01, ^#^*P* < 0.05, and ^##^*P* < 0.01). **(B)** Western blotting analysis of BAX, BCL-2, TP53, and Caspase 3 protein expression using specific antibodies. β-actin was used as a loading control (left side). A histogram showing the statistics of the above mentioned results (right side). All experiments were repeated at least three times with similar results (^*^P < 0.05, ^**^P < 0.01, ^#^*P* < 0.05, and ^##^*P* < 0.01).

To investigate the possible mechanism responsible for the rapid loss of cell viability and apoptosis, we examined the transcription and protein expression levels of the tumor suppressor TP53 and key apoptosis-related genes, including BAX, Caspase 3 and BCL-2, by Q-PCR and western blotting. Q-PCR showed that the overexpression of L-PDCD5 in H293T cells increased the mRNA expression levels of BAX, TP53, and Caspase 3, which were significantly decreased in BCL-2 mRNA transcripts with or without CDDP treatment compared with the empty vector control cells (Figure 8A). Western blotting analysis using specific antibodies against these proteins showed results identical to those of the Q-PCR assay (Figure 8B). Interestingly, the overexpression of L-PDCD5 in cells enhanced the expression levels of BAX and TP53 and suppressed the expression level of BCL-2 independent of Caspase 3. Moreover, an obvious difference was observed in the expression of TP53, BAX, and BCL-2 proteins in the presence of CDDP. Overall, our results indicate that the overexpression of L-PDCD5 leads to the dysregulation of TP53, BAX, and BCL-2 in H293T cells. However, to identify functional differences between L-PDCD5- and L-PDCD10-overexpressing H293T cells, we further investigated key molecules of apoptosis. The Q-PCR results showed that BCL-2 expression was upregulated in the L-PDCD10-overexpressing H293T cells, while BAX expression was downregulated compared with the empty vector control cells (Figure 9A). Moreover, L-PDCD10 overexpression inhibited the expression of the apoptotic factors TP53, BAX, and Caspase 3 after CDDP treatment and reduced the sensitivity of the cells to drugs to cells. Western blotting by using specific antibodies against these proteins was then performed to further confirm the Q-PCR results (Figure 9B). The data showed that the pattern of protein dysregulation is basically consistent with that observed in the Q-PCR data, and overexpression of L-PDCD10 leads to the dysregulation of BAX/BCL-2, increases proliferation and decreases apoptosis in H293T cells.

## Discussion

Jawless vertebrates are significant representative models for investigating the evolution of native and adaptive immunity due to their unique position in the phylogenetic development of chordates. Lampreys and hagfish, which are extant jawless vertebrates, have an alternative lymphocyte-based adaptive immune system that is based on somatically diversifying leucine-rich repeats (LRRs) (32). Animal development is based on multiple cellular functions, and recently, programmed cell death has attracted extensive attention (33), but the lamprey programmed cell death-related PDCD family genes with functional conservation and innovation were unidentified. In the current study, we cloned five cDNA sequences in lampreys that are homologous to PDCD proteins based on the information obtained from the three-generation sequencing library and the primary analysis of ESTs. The NCBI database BLAST search analysis indicated that the nucleotide and deduced amino acid sequences of lamprey PDCD family genes share a high sequence similarity with human PDCD family gene homologs. Based on the above mentioned observations, we named these genes L-PDCD2, L-PDCD4, L-PDCD5, L-PDCD6, and L-PDCD10. The open reading frames of L-PDCD2, L-PDCD4, L-PDCD5, L-PDCD6, and L-PDCD10 contained 1158, 1440, 387, 567, and 630 bp, respectively (Table 2). The analysis of the deduced amino acid sequences indicated that these five L-PDCD proteins contain different domains, phylogenetic analysis revealed that the L-PDCD proteins are localized in different clusters, and the overall evolutionary scenarios of these genes are summarized in Figures 1, 2. In this study, comparative genetic mapping between jawless and jawed vertebrates was conducted to examine the syntenic relationships of PDCD family genes. Unfortunately, the PDCD1 and PDCD7 genes were unidentified in lampreys. PDCD1 is an important immunosuppressive molecule of an immunoglobulin superfamily. This protein is expressed in pro-B-cells and is thought to play a role in their differentiation. Moreover, PDCD1 may also be important in T cell function and contribute to the prevention of autoimmune diseases (6). The PDCD7 gene product is involved in specific apoptotic processes in T-cells (17). T lymphocytes and B lymphocytes in lampreys are not similar to those in humans, suggesting that the L-PDCD1 and L-PDCD7 genes are not expressed during this period.

To gain insights into the tissue distribution of the PDCD genes in lampreys, L-PDCD family genes were detected in most tissues of lampreys by real-time PCR. The transcripts of all characterized L-PDCD family genes, especially L-PDCD4 and L-PDCD5, were less abundant in the lamprey tissues. However, the level of L-PDCD10 expression was greater than that of other genes in most of the tissues. This study aimed to provide improved information regarding the PDCD family genes in lampreys, whose molecular features and functions have not been widely investigated. To achieve these goals, we aimed to confirm whether the L-PDCD family is involved in the immune response against foreign pathogens. Because lamprey blood was removed from the tail and the leukocytes of lamprey were obtained by Ficoll-Paque gradient centrifugation of the blood with lymphocyte separating solution, the primary cell separation of other tissues required trypsinase digestion. To avoid the influence of the L-PDCD family by cellular apoptosis in the process of cell digestion, leukocytes were used to investigate the immune response of the L-PDCD family in the following experiments. Interestingly, except for the L-PDCD4 gene, marked downregulation of the L-PDCD family genes was observed in the examined leukocytes of the Poly I:C-infected lampreys (Figure 3). However, our results also revealed that upregulation of L-PDCD gene expression was observed in leukocytes infected with *S. aureus* and *V. anguillarum* at different time points. A possible reason for the promoter elements of the transcription factor binding site of the L-PDCD gene families are differences in lampreys. The second possibility is that the roles of L-PDCD family genes in immune defense may depend on their interaction molecules. The third possibility is that Gram-negative and Gram-positive bacteria and viral mimics inhibit or interfere with the regulation of cell apoptosis through the L-PDCD family genes with regard to the immune response of lampreys. Further investigations are needed to provide more in-depth analyses of the regulation of apoptosis by L-PDCD family genes and the mechanism of L-PDCD gene family activation in lampreys. Importantly, our findings support a model in which Gram-negative *V. anguillarum*-mediated apoptosis alone induces the oligomerization of L-PDCD5. These results indicate that L-PDCD5 itself exists stably in various tissues of lampreys in the form of a polymer. Because Poly I:C has broad-spectrum antiviral and immunomodulatory functions (34), but its antigen epitope is relatively singular, the GCRV virus was used to stimulate lampreys in this part of the experiment. The GCRV virus, which is the most virulent virus in Reoviridae and aquatic reovirus, substantially endangers freshwater fish cultures (35). However, GCRV stimulation affects the immune regulation of vertebrates, including lamprey (36). Overall, under the stimulation of exogenous substances, such as *V. anguillarum* and GCRV, L-PDCD5 becomes an oligomer and monomer to induce apoptosis (Figure 4C).

Apoptosis is a type of programmed cell death in which dead cells exhibit highly conserved morphological changes, including blebbing, cell shrinkage, nuclear fragmentation, and chromatin condensation (37). Apoptosis can be induced by a variety of means, such as radiation, drugs, growth factor withdrawal, hormones and stress (38–40). Many genes are closely associated with apoptosis regulation. Furthermore, to reveal when this key molecule originates and how it functions in the whole stress signal, we reported a PDCD family gene in this study, called L-PDCD5. However, human PDCD5 is essential for inflammation and cancer through regulating apoptosis (41–44). CDDP is a chemotherapeutic drug used in the clinical treatment of a variety of tumors. CDDP functions by inhibiting DNA replication, leading to cell cycle arrest and apoptosis (45). Human PDCD5 has been identified as a proapoptotic molecule based on its ability to enhance programmed cell death triggered by various stimuli (46–49). Previous studies have shown that rH-PDCD5 plays a significant role in intercellular transport in various cells via a clathrin-independent endocytic pathway that originates from heparan sulfate proteoglycan binding and lipid rafts (30). There is increasing evidence suggesting that exogenous rH-PDCD5 added to culture medium could enhance cellular apoptosis triggered by certain stimuli (50–53). In our study, confocal microscopy showed that when the Alexa 488-labeled rL-PDCD5 protein was added to leukocytes, the rL-PDCD5 protein was transported to the cell membrane, migrated from the cytoplasm to the nucleus, and the chromatin of the nucleus was highly coagulated and marginalized, indicating that excessive expression of L-PDCD5 activates the apoptosis pathway and induces apoptosis. This observation also indicated that the uniqueness of L-PDCD5 function in lamprey leukocytes was verified. In combination with the data shown in Figure 4, the expression level of L-PDCD5 decreased first and then increased after stimulation by pathogens. It is inferred that L-PDCD5 typically decreased at the beginning of stimulation by various pathogens but could accelerate cell apoptosis when the expression of L-PDCD5 recovered or increased with the prolongation of stimulation time. To identify functional differences between L-PDCD5 and H-PDCD5, we focused on the investigation of the L-PDCD5 protein in human cell function. Furthermore, after adding Alexa 488-labeled rL-PDCD5 to MCF-7 cells for 15 min, the protein entered the nucleus by the membrane-cytoplasm-nucleus, indicating that the PDCD5 protein enters the nucleus by endocytosis. Then, during the L-PDCD5 treatment of MCF-7 cells for a long period of time, we observed that the proteins accumulated in the nucleus, and the morphological changes of the cells were obvious. Because the exon 3 deletion of Caspase 3 in the MCF-7 cell line leads to the early termination of the protein translation process, the MCF-7 cell line cannot express the Caspase 3 protein (54). These results also indicate that L-PDCD5 induces the apoptosis of cells in the presence or absence of Caspase 3.

H-PDCD2 was transiently expressed in immature thymocytes and induced apoptosis via BCL-6 regulation (55). The overexpression of H-PDCD5 in response to chemotherapeutic agents promotes tumor cell death and anti-inflammatory effects *in vivo* (56, 57). The H-PDCD10 protein enhances cell proliferation and protects malignant T cells from apoptosis (58). Indeed, we were able to detect L-PDCD5 in the cytoplasm and nucleus of HeLa and H293T cells, whereas L-PDCD2 and L-PDCD10 were localized only in the cytoplasm. Interestingly, our data demonstrated that the transfection of L-PDCD5 into H293T cells changed the biological behaviors of cells, induced cellular apoptosis, inhibited cell growth, and enhanced CDDP sensitivity. The apoptosis assay of L-PDCD2-overexpressing H293T cells showed results identical to those of L-PDCD5-overexpression cells. Conversely, our results showed that the overexpression of L-PDCD10 in H293T cells played a major role in the resistance to cisplatin-induced apoptosis (Figure 7).

Furthermore, the molecular mechanism of L-PDCD5-induced apoptosis was investigated. During the progression of apoptosis, the expression levels of antiapoptotic and proapoptotic proteins are strictly regulated. Caspase 3 is a terminal effector molecule of the apoptosis signal transduction pathway. Numerous studies have demonstrated that H-PDCD5 promotes its cleavage and activation, leading to the occurrence of apoptosis (59, 60). H-PDCD5 causes cell apoptosis by regulating the expression of BCL-2 family members (61). For the TP53 gene, the presence of PDCD5 and drug treatment increases the activity of TP53, thereby increasing the interaction between PDCD5 and TP53 to induce cell apoptosis (62). In this study, the expression levels of BCL-2 family proteins and TP53 were detected by Q-PCR and western blotting analysis. The expression levels of TP53 and BAX were increased, and the expression level of BCL-2 was decreased after 24 h of transfection with the pEGFP-N1-PDCD5 plasmid. The ratio of BAX/BCL-2 was thus increased, and apoptosis is expected to be induced in these conditions. Although Caspase 3 activity was found to be activated by CDDP, only overexpression of L-PDCD5 had little effect on the Caspase 3 pathway at the posttranslational level. In conclusion, the overexpression of L-PDCD5 is mainly involved in the following two signaling pathways: the BAX and TP53 apoptotic pathways.

Before 2005, studies tended to suggest that H-PDCD10 may be an antiapoptotic gene. However, studies in recent years have revealed that H-PDCD10 also has other functions, such as the combination of H-PDCD10 and STK25 under oxidative stress promoting cell apoptosis (63). H-PDCD10 and MST4 work together. In contrast, it can promote cell proliferation (64). Interestingly, in the L-PDCD10-overexpressing H239T cells, we observed that the upregulation of BCL-2 reduced cell apoptosis. Furthermore, the expression of BAX, Caspase 3, and TP53-related apoptotic factors was decreased by reducing the sensitivity of cells to CDDP. Overall, further studies are needed to verify the conditions under which L-PDCD10 functions in cell lines, but it can be inferred that the multiple interacting proteins of L-PDCD10 are the key to its multipotency.

Overall, the identification of the L-PDCD family in the lamprey genome has improved our understanding of the evolution of the L-PDCD gene family. Its tissue distribution and functions in lampreys also provide a new avenue of research for investigating the evolution of their immune system. Furthermore, investigations of the regulation of apoptosis and L-PDCD gene family activation could contribute to improving the understanding of the lamprey immune system and may help to elucidate the innate immune mechanisms of other organisms. Investigations of the significance and specificity of this L-PDCD5 and L-PDCD10 regulatory pathway in biology and disease is an important goal in future studies.

## Data Availability

The raw data supporting the conclusions of this manuscript will be made available by the authors, without undue reservation, to any qualified researcher.

## Ethics Statement

The animal experiments were performed in accordance with the regulations of the Animal Welfare and Research Ethics Committee of the Institute of Dalian Medical University's Animal Care protocol (Permit Number: SCXK2008-0002).

## Author Contributions

QL, YP, and XG designed experiments. XG, JL (flow cytometry detected), and FS (confocal microscope taken a photo) carried out experiments. XG analyzed experimental results. XG and YP wrote the manuscript.

### Conflict of Interest Statement

The authors declare that the research was conducted in the absence of any commercial or financial relationships that could be construed as a potential conflict of interest.

## Supplementary Material

Figure S1 **(A)** Nucleotide and deduced amino acid sequences of lamprey PDCD5. The PDCD5 domain is shown in red. **(B)** SDS-PAGE analysis of the L-PDCD5 protein expressed in *E. coli* BL21. M, low molecular weight protein marker; and Lane 1, purified recombinant L-PDCD5 protein. **(C)** Titer detection of the rL-PDCD5 polyclonal antibody. **(D)** The L-PDCD5 antibody was analyzed by western blotting in the dose-dependent rL-PDCD5 protein. M, prestained protein marker.

Figure S2 Immunofluorescence assays were used to detect the localization of L-PDCD5 in the live cells and supraneural bodies of lampreys. The primary antibody was the rabbit anti-rL-PDCD5 antibody (5.0 μg/mL), and the secondary antibody was an Alexa Fluor 488-conjugated goat anti-rabbit IgG antibody (5.0 μg/mL). The cells were observed and imaged with a Zeiss LSM 780 inverted microscope (Carl Zeiss, Inc.,) and analyzed using Zeiss ZEN LE software (60 × magnification). Scale bars: 5 μm.

Supplemental Video 1 Time-lapse imaging of L-PDCD5 overexpression in living cells.
