# Supplementary figures and images for: The Molecular Evolution and Functional Divergence of Lamprey Programmed Cell Death Genes
Source: Front Immunol. 2019 Jun 20;10:1382. doi: 10.3389/fimmu.2019.01382 (PMC6596451; doi:10.3389/fimmu.2019.01382)

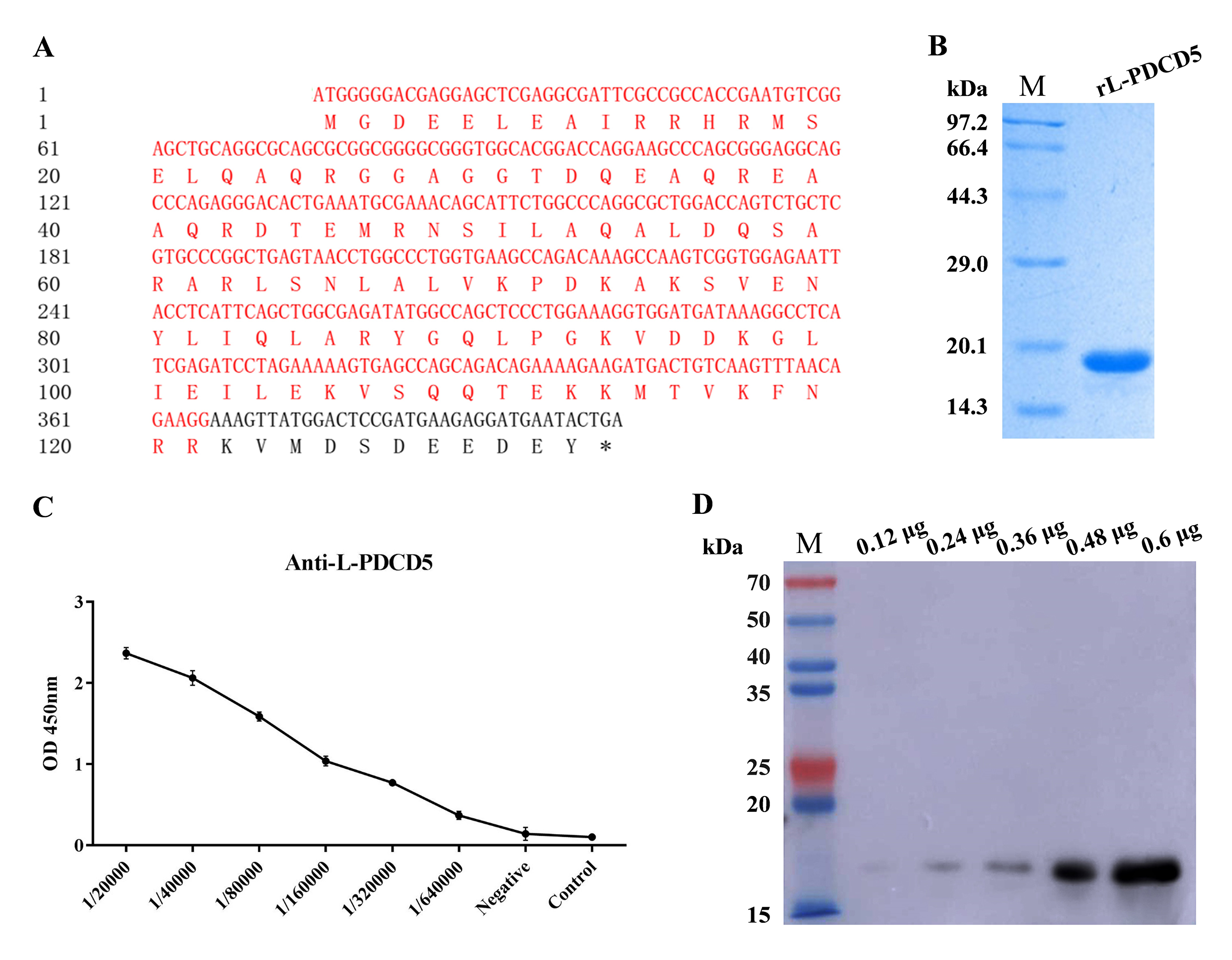

Supplement: Figure S1 — (A) Nucleotide and deduced amino acid sequences of lamprey PDCD5. The PDCD5 domain is shown in red. (B) SDS-PAGE analysis of the L-PDCD5 protein expressed in E. coli BL21. M, low molecular weight protein marker; and Lane 1, purified recombinant L-PDCD5 protein. (C) Titer detection of the rL-PDCD5 polyclonal antibody. (D) The L-PDCD5 antibody was analyzed by western blotting in the dose-dependent rL-PDCD5 protein. M, prestained protein marker. [file Image_1.JPEG]

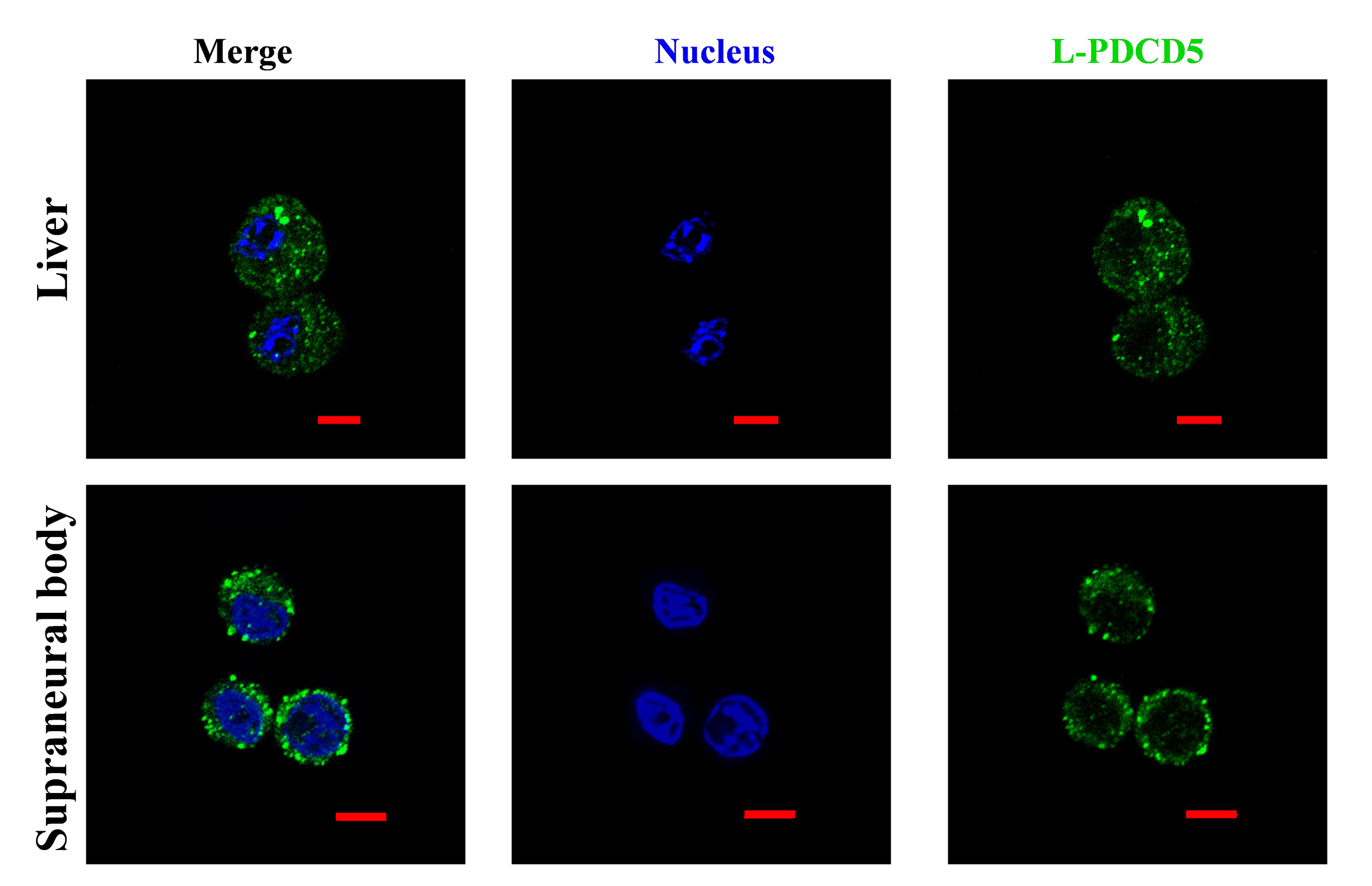

Supplement: Figure S2 — Immunofluorescence assays were used to detect the localization of L-PDCD5 in the live cells and supraneural bodies of lampreys. The primary antibody was the rabbit anti-rL-PDCD5 antibody (5.0 μg/mL), and the secondary antibody was an Alexa Fluor 488-conjugated goat anti-rabbit IgG antibody (5.0 μg/mL). The cells were observed and imaged with a Zeiss LSM 780 inverted microscope (Carl Zeiss, Inc.,) and analyzed using Zeiss ZEN LE software (60 × magnification). Scale bars: 5 μm. [file Image_2.JPEG]
